# The effects of spinal dysraphism on the quality of life of pediatric patients and their families

**DOI:** 10.1080/10790268.2025.2510721

**Published:** 2025-06-09

**Authors:** Remi M. Rufus-Toye, Sabba Hussain, Yael Gelfer, Donato Giuseppe Leo, Anna Bridgens

**Affiliations:** 1 Department of Trauma and Orthopaedics, St George’s University Hospitals NHS Foundation Trust, London, UK; 2 Institute of Medical and Biomedical Education, St George’s University of London, London, UK

**Keywords:** Spinal dysraphism, Spina bifida, Meningocele, Myelomeningocele, Lipomyelomeningocele, Quality of life

## Abstract

**Introduction:**

Spinal dysraphism describes a spectrum of conditions resulting from aberrant neural tube closure during the first trimester of fetal development. Consequent neurological deficits may lead to a range of musculoskeletal abnormalities requiring orthopedic intervention. This study sought to evaluate the impact of spinal dysraphism on patients and their families.

**Methods:**

Free text questionnaires/interviews were collected from parents. Quantitative questionnaires were collected from children using an emoji response system. These assessed contentment in activities of daily living. Qualitative data were analyzed via thematic analysis. Descriptive statistics were applied to quantitative data.

**Results:**

In total 32 responses (13 patients and 19 carers) were collected and analyzed. The median age of patients was 10 years (IQR = 3), with a sex distribution of 7 boys and 6 girls. Overall, 3 key themes were identified: (i) Independence, (ii) Mental Health, (iii) Family Impact, each with corresponding subthemes. Quantitative data corroborated these themes well, highlighting impacts on activities of daily living in the majority of domains assessed.

**Conclusion:**

Spinal dysraphism has a multifactorial impact on patient and family quality of life spanning the societal, mental, and physical domains. These findings will be included in ongoing work to create a set of core outcomes for use in the clinical and research settings.

## Introduction

Spinal dysraphism (SD) describes a spectrum of conditions resulting from aberrant closure of the neural tube during the first trimester of fetal development. This results in either an open (aperta) or closed (occulta) defect of the spine. The incidence of all forms of SD is estimated to be up to 8 cases per 1000 live births in the global north (1, 2). The underlying causes are not fully understood but entail an interaction between genetic, epigenetic, and environmental factors (3, 4). Folic acid insufficiency is a major risk factor and its supplementation before and until the end of the first trimester of pregnancy reduces occurrence significantly (5).

The resulting clinical presentation depends upon the nature of the defect (meningocele, myelomenigocele or lipomyelomeningocele) and its neurological level. The spectrum of neurological involvement impacts urological, neurological and musculoskeletal systems and children can have bladder and bowel dysfunction, reduced sensation and altered motor function, hydrocephalus, Chiari malformations, and tethered cord (6, 7). The resultant muscular imbalances can lead to secondary orthopedic complications such as hip dislocations, tibial torsion, and various foot deformities that are amenable to orthopedic intervention (8).

Significant variation exists in both practice and guidelines for orthopedic intervention in spinal dysraphism (9, 10). Comparisons between these differing practices is made challenging due to the lack of an agreed upon core outcome set for the orthopedic management of spinal dysraphism (11). In recent years there has been movement toward the development of core outcome sets for other pediatric orthopedic conditions (12–14).

A critical step in developing a set of core outcomes is to gain the insight of all key stakeholders, including patients and understanding the factors which are important to quality of life (QoL) (15–17). The objectives of this study were to capture the physical, emotional, and social impacts of spinal dysraphism on children aged 0–16 and their families. Providing this insight will facilitate the creation of patient centered, as opposed to the existing clinician centered, outcome measures. This work is a part of a broader effort to develop orthopedic core outcome sets for SD (18).

## Methods

### Study design

A mixed methods study was designed and employed to capture the factors deemed important to patients and families with SD. Parental/carer free text questionnaires/interviews captured proxy measures of these factors. To improve the robustness of the results, quantitative questionnaires were collected from children with SD. This modality factored in the restrictions introduced by young age.

### Sampling and eligibility

Patients aged 16 years and under with a diagnosis of SD under the care of a tertiary multi-disciplinary SD clinic were eligible for recruitment. The multi-disciplinary team consisted of neurosurgeons, orthopedic surgeons, physiotherapists and clinical nurse specialists. Participants were recruited from clinics at 2 London tertiary centers. A purposive sample of 13 children and 19 parents/carers was deemed adequate to reach saturation. Within the context of qualitative research, saturation refers to the point at which no new codes or themes emerge, even as the sample size increases (19).

### Parental/carer questionnaire design

The parental/carer questionnaire collected both participants’ demographics and responses to a free text questionnaire designed to capture the elements of daily life that the participant felt were impacted by SD.

Parental/carer questionnaires were designed via an iterative process by one of the authors (AB) until it was agreed that the survey captured enough breadth and detail to appreciate a patient/carer centered perspective on QoL (supplementary figure 1).

Based on family preference, 4 structured interviews were conducted in place of free text responses. The questionnaire formed the basis of this interview.

### Children’s questionnaire design

The children’s questionnaire was developed taking into account patient reported outcomes that are commonly assessed in clinical practice (20), whilst also considering the possible impact of SD on their activities of daily living (21). Each item of the questionnaire was scored using a 3-point rating scale, where 1 is the lowest possible score and 3 is the highest. In order for the questionnaire to be easily understood by young children, the numeric value of the responses was substituted with an emoji (sad face = 1, neutral face = 2, happy face = 3). The use of the emoji to simplify patient reported outcome questionnaires for children has been previously employed (22).

The end of the survey contained a free text element for those who were able and wished to provide additional comments. These were filled in either by the patient, the parent or carer (supplementary figure 2).

### Confidentiality and consent

All participants were given a participant information sheet which highlighted the aims, objectives, and voluntary aspect of participation. For younger patients, consent was sought from parents. However, the voluntary nature of participation was highlighted to minors and assent sought. Agreement to participate was accepted as consent.

### Ethical approval

This project was prospectively registered (AUDI004001). On consultation with the institutional research and development department, this project was deemed a service evaluation thus not requiring ethical approval. No patient identifiable information was collected.

### Data security

Electronic copies of the paper questionnaires were stored on a password encrypted computer using an Excel spreadsheet (Microsoft, Seattle, WA). Captured data was anonymized and no patient information was stored outside of secure hospital IT systems. Data access was restricted to the immediate research team on an as-needed basis.

### Data analysis and statistics

The qualitative aspect of the data underwent thematic analysis (23, 24). Analysis was performed using an Excel spreadsheet (Microsoft, Seattle, WA, 2016). Analysis was undertaken by a member of the research team, an academic clinician with formal postgraduate education in mixed methods research (RRT). Immersion in the data corpus was achieved via multiple readings. The data were coded using an inductive approach to minimize bias from the primary author’s clinical perspective. Likewise, themes were cultivated using a semantic approach. The themes defined were reviewed independently by 2 authors (RRT and SH).

The quantitative aspects of the data analysis and visualization were performed using R version 4.3.1 (R Core Team, Vienna, Austria, 2023) and RStudio Version 2023.06.1 (RStudio Team, Vienna, Austria, 2023). The measures of central tendency and spread presented are the median and interquartile range (IQR) respectively.

The qualitative and quantitative arms of the study were triangulated to evaluate convergence, complementation, disagreement, and silence.

## Results

In total 32 responses (13 patients and 19 carers) were collected and analyzed. The median age of patients was 10 years (IQR = 3). The sex distribution was 7 boys and 6 girls.

### Thematic analysis

**Figure 1:**
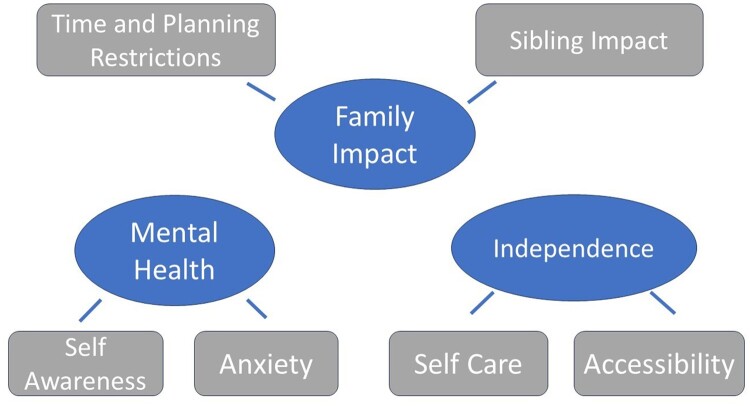
A schematic of the themes and subthemes that were generated from coding.

The 3 key themes that emerged from the thematic analysis were independence, mental health, and family impact. Each of these key themes generated corresponding sub-themes (Fig. 1). The themes are elaborated below using verbatim quotes.

### Independence

Families expressed limitations on the independence of their children. The domains of these restrictions are reflected through the subthemes of (a) Self-care and (b) Accessibility.

#### Self-care

Parents expressed the need for them to be heavily involved in the daily self-care of their children. The levels of requirement included basic activities of daily living as well as requirements secondary to the pathology of SD such as bladder care.

… parent needs to do his catheter, support him eating breakfast/having a drink, get into the car and then helping him to walk to school. (Parent 1)

The significant care needs of patients entailed a constant necessity for a parent’s presence. When referring to activities that their child could not complete without help, Parent 1 reported:

Getting dressed, complete self feeding, brushing own teeth. It means he needs an adult around always … . (Parent 1)

The amount of dependence that children with SD had on their parents was often source of anxiety about the future. Parents expressed a sentiment of fear regarding their child’s ability to take care of themselves in the future. This was in the context of the future loss of capacity of a parent to look after their child. When asked their biggest fear, parents responded:

Her safety/security when my husband and I are no longer around to coordinate [and] support her. (Parent 8)

Caring for her if one of her parents gets ill. (Parent 5)

Additionally, Parent 6 expressed that their biggest fear for the future was:

Not being independent or being able to organise things himself. Obviously, I will always be there for him but would be nice for him to not need me so much as an adult. (Parent 6)

#### Accessibility

The mobility status of their children was reported as source of difficulty for families. These restrictions were experienced in environments that would have significant impacts on families such as their homes.

[It’s] stressful [to] get her up 4 flights of stairs to [the] flat especially with [a] wheelchair on a long or bad day. (Parent 3)

However, the restrictions imposed upon children were found to be secondary to poor access outside of the home.

One significant impact on the family is very limited access to after school clubs and holiday provisions (as H needs a 1–2–1 and many clubs aren’t set up for him). This impacts my ability to work and H to access extracurricular activities alongside his peer group. (Parent 4)

Dependence on others to achieve tasks and take part in activities was reported as a source of frustration for patients. This dependence created a challenging barrier to interacting with peer groups. When explaining why their child would become frustrated, Parent 8 reported:

Once in a while when she has to wait for me to be available to do something eg. reach a high shelf. (Parent 8)

Concerning limitations to their child taking part in activities with friends, Parent 8 reported:

She is not able to just go and meet up with friends. She needs help to get to places. (Parent 8)

Additionally, Parent 4 reported that their child was:

… frustrated that he is not able to keep up with his friends when playing in the playground during PE lessons. (Parent 4)

Parent 4 communicated how uncertainty as to whether society would create an enabling environment for their child was a source of concern for the future.

My biggest fear is whether H will get the support he needs to lead a full life – will he have the same access to jobs and further learning as his siblings, will he be included in friendship groups? (Parent 4)

### Mental health

SD had an impact on the mental health of patients and their families. The impacts upon mental health are expressed via 2 sub themes; (a) Anxiety, (b) Self Awareness.

#### Anxiety (parent and patient)

Parents expressed the concerning levels of anxiety that their children display. The hospital presented a source of this anxiety in more than once case. Additionally, parents reported feeling unequipped and unsupported when managing these types of difficulties.

He feels anxious and worries quite a lot especially about coming to hospital appointments and whether he’ll need more surgery in the future. I struggle to know how to reassure him whilst also being honest … We would love more support around how to help H with his emotional challenges and his anxiety … . (Parent 4)

… it’s very hard dealing with everything, he’s very clingy and hates [the] hospital. (Parent 9)

Anxiety was not solely felt by patients. Parents reported that the uncertainty regarding the trajectory of their child’s illness was a source of anxiety for them.

As parents we are worried about her condition. Anytime it can change … . (Parent 5)

#### Self-awareness

A sub theme of self-awareness was highlighted via discussion of the understanding of one’s condition and the barriers imposed by wider society. Parents worried that their child’s growing appreciation of the differences between themselves and others would have an impact upon their child’s mental wellbeing.

My biggest fear about Y is how we can support him to build a strong and high self-confidence … and be a part of the community without close support. (Parent 11)

H's mental health is a concern too as he starts to become aware of the differences between him and his peer group. (Parent 4)

As this process unravels, parents conveyed a desire to help their children with reaching a place of acceptance.

The main concern … is to support Y understanding and accepting his illness. (Parent 11)

However, the ability to achieve this is complicated by the stigma that patients with SD may experience. When asked about their biggest fear about their child’s future one parent responded:

That he won’t have a normal life and other people will make fun of him … . (Parent 9)

### Family impact

Respondents expressed how the significant amounts of care that their children need and the regimented nature to which this care must be delivered imposes significant restrictions on family life. This includes impact on time with friends and family. Respondents reported that these impacts are keenly felt by siblings. The theme of family impact is conveyed via the subthemes of; (a) Time and Planning Restrictions, (b) Impact on Siblings.

#### Time and planning restrictions

The impact on family activities is multifactorial. Firstly, extra time must be factored into family planning to facilitate care routines of children with SD.

We do have to factor in extra time for her routines. She can make herself some snacks but less than other 11-year-olds. (Parent 8)

In addition, parents conveyed an inability to be spontaneous. Restrictions on time and spontaneity placed restrictions on the activities that families can take part in.

We just cannot ever be spontaneous. Everything in my life and hers must be planned out. (Parent 8)

We have very limited activities as a family which affects the sibling’s wellbeing. (Parent 11)

The result of these restrictions and adaptations is felt by the entire family and is described as tiring:

It can be very tiring and has changed our daily functioning. Everyone had to adapt to accommodate him. It also impacts on what we choose to do as a family. (Parent 1)

However, this impact is not felt in the same way by all families. Some expressed an adjustment to a new normality. When asked about the effects of their child’s condition on the family, Parent 6 reported:

Doesn’t affect us as J has always been the same so just a part of our lives. (Parent 6)

#### Impact on siblings

Parents reported that siblings of children with SD experience restrictions on the experiences they can take part in with their families.

Her brother is more impacted when we are on days out or wanting to visit places with poor access. (Parent 16)

As well as restrictions on the types of activities that the family can partake in, parents also reported having less time to spend with their other children.

The main impact is on the time that we can spend with our other children. (Parent 4)

Finally, the time and attention required for the care of children with SD has an impact on the friendships of their siblings.

It can be hard for them [siblings] to have friends over or have sleepovers as we need to do Hs bowel washouts in the evening. (Parent 4)

### Quantitative analysis

**Figure 2:**
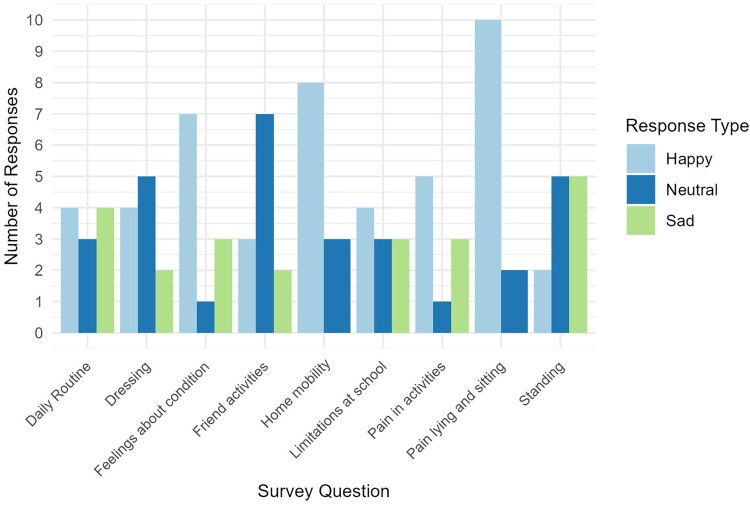
Bar chart demonstrating the responses of patients to questionnaires. The full text prompts, which are abbreviated on the *x*-axis, are available in supplementary material 3.

The survey responses collected from patients for the quantitative arm of the study are presented in Fig. 2.

### Activities of daily living

Respondents were asked to subjectively assess their degree of contentment with activities of daily living using a 3-point rating scale. To facilitate accessibility to young participants numbers were replaced with emojis. A minority of respondents (41.7%, *n* = 5, Fig. 2) responded with a happy emoji when asked to consider the statement “I can do most of my daily routine without assistance”. Likewise, 41.7% (*n* = 5, Fig. 2) responded with a happy emoji when asked “I can dress myself”.

Regarding participation in activities with friends, 4 patients (30.8%, Fig. 2) reported a happy emoji in response to “I can participate in all the activities my friends do”. A total of 5 patients (45.5%, Fig. 2) responded with a happy emoji to “I have no limitations when I am at school”.

### Mobility

Patients were asked questions to subjectively assess their mobility status. A total of 3 (23.1%. Fig. 2) patients responded with a happy emoji when asked “I can stand with no problem?”. However, the majority of patients (75.0%, *n* = 9, Fig. 2) responded with a happy emoji to “I can move around the house without help”.

### Pain and perception of condition

When asked to consider the statement “I feel no pain when I lie down on a bed/sit on a chair”, 11 patients (84.6%, Fig. 2) responded with a happy emoji. No patients responded with a sad emoji. The majority of patients (60.0%, *n* = 6, Fig. 2) responded with a happy emoji to the statement “Pain/discomfort stops me from doing the things I want to do”.

In response to the statement “I feel okay with my condition”, 60.0% (*n* = 6, Fig. 2) of patients responded with a happy emoji. A total of 3 (30.0%, Fig. 2) patients responded with a sad emoji.

## Discussion

The findings of this study reveal that patients with SD experience significant effects on their independence and mental health, which in turn affect the entire family unit. These elements interact to create unique challenges. These results align with previous literature showing impacts on independence, family dynamics, and peer interactions (25, 26). This study highlights how children’s experiences manifest as anxiety.

Parents reported significant limitations on their children’s independence, including needing help with daily activities such as dressing. The results show dependence on parents to fulfill additional needs like accessing objects and leaving the house to meet friends. Challenges also extended outside the home, including limited access to activities like afterschool clubs.

Patients corroborated these findings. In total, 5 patients (41.7%, Fig. 2) responded with a happy emoji to the statement, “I can do most of my daily routine without assistance,” and 5 (45.5%, Fig. 2) did so for, “I have no limitations when I am at school”. Families noted SD’s impact on family time and planning, limiting spontaneous activities and siblings’ participation in certain activities.

A strong interaction emerged between themes of independence and family impact. The frequent and regimented dependence on parents to assist children with SD in their activities of daily living was expressed as causal factor to constrained family spontaneity and reduced parents’ time with other children.

Mental health impacts were linked to children’s anxiety and growing self-awareness. Anxiety often stemmed from hospital-related experiences and uncertainties about the future. Parent 4 described their child’s frustration at being unable to keep up with peers. In total, 60% (*n* = 6, Fig. 2) of patients responded positively to, “I feel okay with my condition”. This suggests children may accept their condition while still feeling frustration and anxiety, which could be mitigated by improved societal accessibility (27).

Pain was rarely reported. When asked about pain during rest, 84.6% (*n* = 11, Fig. 2) responded positively, potentially due to altered sensation in the lower limbs (28).

### Limitations and future work

This study’s mixed-methods approach provided rich insight into how SD impacts quality of life, but sampling had limitations. Purposive sampling from two English tertiary centers limits generalizability, and the small sample size risks limiting representation of the population. However, thematic saturation was achieved, as discerned by the point at which no further codes were generated. Future work focusing on the quantitative aspect of this study may facilitate an increased sample size. Correlating this with clinician-based assessment of functional status may provide novel insights.

Most qualitative data came from parents. Although the children’s questionnaire aimed to capture qualitative data, their young age and low response rate limited this. Proxy measures of QoL were strengthened by triangulating children’s quantitative data with parents’ qualitative data.

This study sheds light on the multifaceted impacts of SD on patients and families. Future multi-center studies outside the UK would enhance generalizability. Insights from this study will inform the development of a core outcome set for the orthopedic management of spinal dysraphism (18).

## Conclusion

This study has identified the unique needs of children and their families with SD that include social, psychological, and emotional factors. This is in addition to the more obvious physical needs of the child, such as the urological, neurological, and musculoskeletal aspects. This study identified key outcomes of importance to patients and families with SD helping to develop a core outcome set in the orthopedic management of SD.

## Supplementary Material

Childrens questionnaire.pdf.pdf

Description of Supplementary Material.docx

Supplementary Material 3.docx

parents questionnaire copy.pdf.pdf

## Data Availability

The full data set is available upon reasonable request to the corresponding author.
