# Supplementary material for: The effects of spinal dysraphism on the quality of life of pediatric patients and their families
Source: J Spinal Cord Med. 2025 Jun 9;49(3):536–42. doi: 10.1080/10790268.2025.2510721 (PMC13123046; doi:10.1080/10790268.2025.2510721)
Supplement: Childrens questionnaire.pdf.pdf [file YSCM_A_2510721_SM4100.pdf]

Patient-Reported Outcomes in Spina Bifida and related Orthopaedic management

Children Questionnaire (8-11 years old)

Dear Parent/Guardian,

We are studying how spina bifida and its related orthopaedic (bone and joint and mobility) conditions affect your child's daily living activities. Part of this study is to understand how young people living with spina bifida are limited during their daily activities. The following short questionnaire will help them communicate how they feel.

Many thanks for your help.

The Research Team

INTRODUCTION

In the next few pages, we will ask you questions about how your condition limits you in your daily activities. Please choose the smiley face that best matches your answer.

ABOUT YOU

Your age………..

Your gender

Male **Female** **Prefer not to say**

**QUESTIONS**	
**Questions**	**Sad Ok-ish Happy** 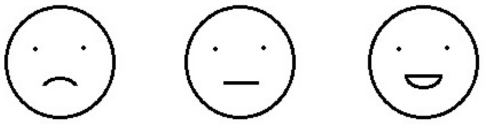
1. I can dress myself.	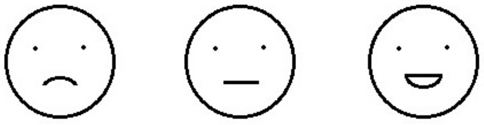
2. I can move around the house without anyone helping me.	

	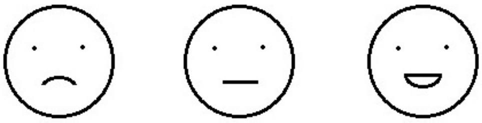
3. I can do most of my daily routine without any assistance.	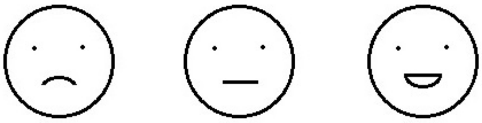
4. I can participate in all the activities my friends do.	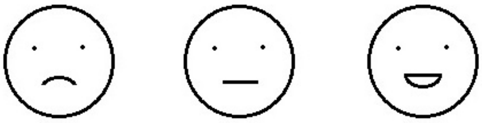
5. I feel no pain when I lie down on a bed/sofa or sit on a chair.	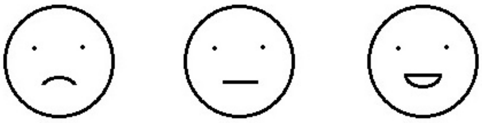
6. I can stand with no problem.	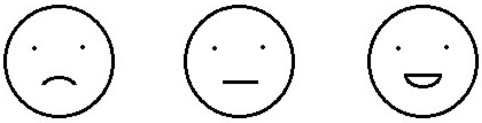
7. Pain/discomfort stops me doing things I want to do	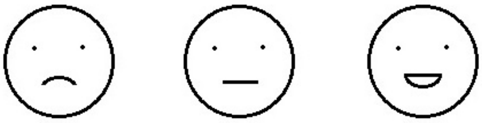
8. I feel ok with my condition.	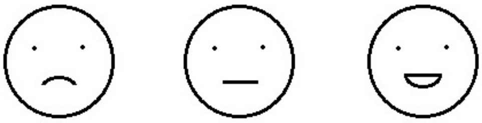

9. I have no limitations in when I am at school.	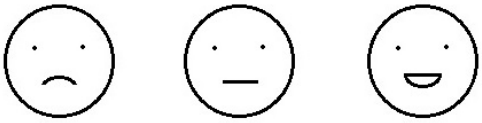

Can you tell us about any other limitations/feelings you have about your condition?

We would like to know if you have any other difficulties in doing activities you like to do or if you feel sad about your condition. For example, you may worry about something related to your condition, or feel unhappy about some aspects of your treatment or upset that you can not always join in with your friends.

……………………………………………………………………………………

……………………………………………………………………………………

……………………………………………………………………………………

……………………………………………………………………………………

……………………………………………………………………………………

……………………………………………………………………………………

……………………………………………………………………………………

……………………………………………………………………………………

……………………………………………………………………………………

……………………………………………………………………………………

……………………………………………………………………………………

…………………………………………………………

YOU HAVE NOW FINISHED. THANK YOU.

PLEASE SEND THIS QUESTIONNAIRE BACK TO US.
