# Supplementary material for: The effects of spinal dysraphism on the quality of life of pediatric patients and their families
Source: J Spinal Cord Med. 2025 Jun 9;49(3):536–42. doi: 10.1080/10790268.2025.2510721 (PMC13123046; doi:10.1080/10790268.2025.2510721)
Supplement: Description of Supplementary Material.docx [file YSCM_A_2510721_SM4099.docx]

**Description of Supplementary Material**

Supplementary material 1: Copy of the parental questionnaires

Supplementary material 2: Copy of the children’s questionnaires

Supplementary material 3: The full text prompts to the questionnaire responses that are represented in the x axis of figure 2.
