# Supplementary material for: The effects of spinal dysraphism on the quality of life of pediatric patients and their families
Source: J Spinal Cord Med. 2025 Jun 9;49(3):536–42. doi: 10.1080/10790268.2025.2510721 (PMC13123046; doi:10.1080/10790268.2025.2510721)
Supplement: Supplementary Material 3.docx [file YSCM_A_2510721_SM4098.docx]

| **Abbreviation** | **Full Prompt** |
| --- | --- |
| Daily Routine | "I can do most of my daily routine without assistance" |
| Dressing | "I can dress myself" |
| Feelings About Condition | "I feel okay with my condition" |
| Friend Activities | "I can participate in all the activities my friends do” |
| Home Mobility | “I can move around the house without help” |
| Limitations at School | “I have no limitations when I am at school” |
| Pain in Activities | “Pain/discomfort stops me from doing the things I want to do” |
| Pain Lying and Sitting | “I feel no pain when I lie down on a bed/sit on a chair” |
| Standing | “I can stand with no problem” |
