# Supplementary material for: The effects of spinal dysraphism on the quality of life of pediatric patients and their families
Source: J Spinal Cord Med. 2025 Jun 9;49(3):536–42. doi: 10.1080/10790268.2025.2510721 (PMC13123046; doi:10.1080/10790268.2025.2510721)
Supplement: parents questionnaire copy.pdf.pdf [file YSCM_A_2510721_SM4097.pdf]

Patient-Reported Outcomes in Spina Bifida and related Orthopaedic management

Parents' Questionnaire

Dear Parent/Guardian,
We are studying how spina bifida and its related orthopaedic conditions impact your child's daily living activities. Part of this study is to understand your feelings about being a parent of a child with spina bifida, and how this condition affects you and your family. The questions that follow aim to collect your views on this topic.

Many thanks for your help.
The Research Team

ABOUT YOU

Before you begin, we would like to ask you a few general questions about yourself.

What is your **gender**? Male Female Prefer not to say

How old are you? _______ (years)

What is the highest **education** you received?

- Primary school
- Secondary school
- Tertiary – college or university

How many children do you have? _____________

INSTRUCTIONS

In the next few pages, we would like to ask you some questions about how your child's condition is affecting what s/he is able to do day-by-day and how his/her condition is affecting you and your family. Please answer the **open questions** giving as much details possible, and complete the **multiple-choice questions** circling the score that best matches your feeling.

Please try to answer **<u>all the questions</u>**.

QUESTIONS

Is your child:

a) an independent walker

b) able to walk with assistance of frame

c) unable to walk/ a non walker

Does your child have splints/orthosis: YES / NO

If yes, what type?

Daily Limitations

1. Does his/her condition affect the family daily schedule (e.g. getting up and dressed; going to school/work)? And how?

…………………………………………………………………………………

…………………………………………………………………………………

…………………………………………………………………………………

…………………………………………………………………………………

…………………………………………………………………………………

…………………………………………………………………………………

………………………………

2. Is there any activity s/he cannot do (e.g. getting up and dressed on their own; sitting on a chair)? And how does it affect yours and his/her daily living?

…………………………………………………………………………………

…………………………………………………………………………………

…………………………………………………………………………………

…………………………………………………………………………………

…………………………………………………………………………………

…………………………………………………………………………………

………………………………

3. Does your child feel pain in his/her back/hip/knee/feet? If yes, please tick the relevant boxes and please explain when s/he feels it (e.g. while standing; when sitting; etc.) in the “additional details” section below.

	**Never**	**Rarely**	**Sometimes**	**Often**	**Always**
**Back**					
**Hips**					
**Knees**					
**Ankle/Foot**					

Additional details:

…………………………………………………………………………………

…………………………………………………………………………………

………………………………………………………………………………………

………………

Social Life

4. Is your child’s condition limiting his/her ability to take part in any activity with his/her friends?

- Never
- Rarely
- Sometimes
- Often
- Always

General Health

5. Does your child suffer from any other medical condition apart from spina bifida (e.g. hydrocephalus, shunt, incontinence)?

- Yes
- No

additional details:

………………………………………………………………………………………

………………………………………………………………………………………

…………

Emotional Barriers

6. How does your child feel about his/her condition?

- Sad
- Afraid
- Worried
- Frustrated
- None of the above
- Other

Family Impact

7. How is your child's condition affecting you and your family?

……………………………………………………………………………………

……………………………………………………………………………………

……………………………………………………………………………………

……………………………………………………………………………………

……………………………………………………………………………………

……………………………………………………………………………………

………………………………

…………………………………………………………………………………

…………………………………………………………………………………

…………………………………………………………………………………

…………………………………………………………………………………

…………………………………………………………………………………

…………………………………………………………………………………

………………………………

8. What is your biggest fear about your child's future?

…………………………………………………………………………………

…………………………………………………………………………………

…………………………………………………………………………………

…………………………………………………………………………………

…………………………………………………………………………………

…………………………………………………………………………………

………………………………

9. Is there anything else you would like to add? For example, anything else which you feel is of particular importance or significance at the moment and which you think as researchers, we should know about?

…………………………………………………………………………………

…………………………………………………………………………………

…………………………………………………………………………………

…………………………………………………………………………………

…………………………………………………………………………………

…………………………………………………………………………………

………………………………

10. Do you feel that the medical team understand you and your child's needs and do everything they can to address them?

…………………………………………………………………………………

…………………………………………………………………………………

…………………………………………………………………………………

…………………………………………………………………………………

…………………………………………………………………………………

…………………………………………………………………………………

………………………………

YOU HAVE NOW FINISHED. THANK YOU.

PLEASE SEND THIS QUESTIONNAIRE BACK TO US.
